# Impact of Active Ankle Movement Frequency on Velocity of Lower Limb Venous Flow following Total Hip Arthroplasty

**DOI:** 10.1155/2016/7683272

**Published:** 2016-11-23

**Authors:** Tsutomu Nakayama, Sachiyuki Tsukada, Takayuki Hiyama, Tatsuya Yamada, Naoyuki Hirasawa

**Affiliations:** ^1^Department of Rehabilitation, Hokusuikai Kinen Hospital, Ibaraki, Japan; ^2^Department of Orthopaedic Surgery, Hokusuikai Kinen Hospital, Ibaraki, Japan; ^3^Department of Clinical Laboratory, Hokusuikai Kinen Hospital, Ibaraki, Japan

## Abstract

*Background*. Although active ankle movement plays a predominant role in mechanical thromboprophylaxis following total hip arthroplasty (THA), the most effective frequency of movement remains unclear.* Materials and Methods.* In 29 consecutive patients undergoing THA, the velocity of blood flow in the profunda femoris was measured after various frequencies of ankle movement two days after THA using a pulse wave Doppler ultrasound system. To test the interobserver reliabilities for the velocity measured with Doppler ultrasound system, the intraclass correlation coefficient was calculated based on the measurement in 10 limbs of healthy volunteers.* Results.* At 0, 1, and 2 minutes after ankle movement, the velocity after movement at 60 contractions per minute was significantly faster than that after movement at 40 or 80 contractions per minute (*p* = 0.0007, repeated-measures analysis of variance). The intraclass correlation coefficient score in two investigators was 0.849 (95% confidence interval, 0.428 to 0.962).* Conclusions.* Active ankle movement at 60 contractions per minute is recommended in patients receiving THA to obtain optimal venous blood flow.

## 1. Introduction

Venous thromboembolism (VTE) is the most important preventable cause of mortality in patients following total hip arthroplasty (THA) [1, 2]. Active movement of the ankle can increase venous blood flow of the limb following THA [3] and has been proposed as an effective means of preventing VTE [4].

Kagaya investigated the relationship between frequency of ankle movement and venous blood flow in six healthy women and concluded that ankle movement at a rate of 60 contractions per minute was most effective to obtain a high velocity of venous blood flow [5]. The most effective frequency of ankle movement for patients following THA remains unclear because venous blood flow of the limb following THA differs from healthy limb [6].

We investigated patients undergoing THA to assess the relationship between frequency of ankle movement and venous blood flow. The hypothesis of this study was that the ankle movement at 60 contractions per minute would increase venous blood flow more effectively than that at 40 or 80 contractions per minute in patients undergoing THA.

## 2. Materials and Methods

The study was approved by our institutional review board, and patients provided written informed consent. This study was performed at the rehabilitation department of a single private hospital.

Patients scheduled for unilateral THA from March 2015 to May 2015 were eligible for inclusion. Patients scheduled for revision THA or simultaneous bilateral THA and with peripheral circulation insufficiency in the lower limb were excluded.

### 2.1. Intervention and Outcome

The study interventions were three different frequencies of active ankle movement: 40 contractions per minute, 60 contractions per minute, and 80 contractions per minute. The primary outcome was the mean velocity of blood flow in the profunda femoris measured two days after surgery using a pulse wave Doppler ultrasound system (APLIO 400 TUS-A400; Toshiba Medical Systems Corporation, Tochigi, Japan).

The patients received instruction regarding the ankle movement used in the study protocol one day before surgery. Baseline measurement of venous flow in the profunda femoris was obtained on the same day as instruction was given.

The patients were instructed to move the ankle of the affected side in time to a metronome. The patients were in the supine position with the knees and hips in a neutral position and instructed to perform ankle plantar flexion and dorsiflexion over the full range of motion.

All measurements of blood flow velocity were performed by a single experienced clinical laboratory technician (TH). The measurement site of the profunda femoris was 3 cm proximal from the bifurcation point of the common femoral vein.

**Figure 1:**
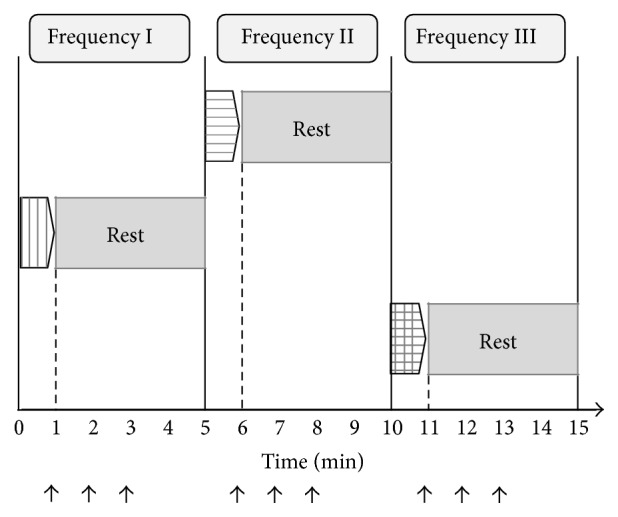
Protocol for active ankle movement at three frequencies and measurement of blood flow velocity at the profunda femoris. After one minute of active ankle movement, the velocity of venous blood flow was measured three times (black arrows indicate the point of measurement: (1) just after movement, (2) 1 minute after movement, and (3) 2 minutes after movement). The order of the three different frequencies (40, 60, and 80 contractions per minute) was determined by drawing lots. Four minutes after the first frequency of ankle movement, another frequency of active ankle movement was performed, and similar measurements were repeated.

The order of the three different frequencies of ankle motion was determined by drawing lots just prior to the measurement. The measurement protocol is shown in Figure 1. The active ankle movement at the predetermined frequency was performed for one minute. The venous blood flow velocity in the profunda femoris was measured by Doppler ultrasound just after 1 minute and 2 minutes after 1 minute of ankle movement. Four minutes after the first set of ankle movement and measurements, another frequency of active ankle movement was performed, and the velocity of venous blood flow was measured in the same manner. Again, 4 minutes after the second set of ankle movement and measurements, active ankle movement was performed at the third frequency, and the velocity of venous blood flow was measured in the same manner.

### 2.2. Surgery, Rehabilitation, and Postoperative Medications

All patients received general anesthesia without any epidural injection. Surgery was performed using a minimally invasive anterolateral approach without detachment of muscle or tendon with the patient in the lateral decubitus position [7]. All surgeries were performed or supervised by a high-volume hip surgeon (NH).

To prevent VTE, intraoperative calf bandage and postoperative intermittent pneumatic compression until 09:00 one day after surgery were performed. Rehabilitation was started one day after surgery. All patients were screened to detect deep vein thrombosis using a pulse wave Doppler ultrasound system on the morning after THA.

No routine medication was given to prevent VTE. Antibiotic prophylaxis with a first-generation cephalosporin (Cefamezin [cefazolin]; Astellas, Tokyo, Japan) was administered intravenously perioperatively and every 8 hours for the first 48 hours after surgery. From the day after surgery, oral nonsteroidal anti-inflammatory drug (4 mg of lornoxicam, Lorcam; Taisho-Toyama, Tokyo, Japan) was administered three times a day.

### 2.3. Statistical Analysis

For primary outcome, we used repeated-measures analysis of variance with Scheffé's post hoc test to assess differences between the three groups. A two-sided *p* value of <0.05 was considered significant.

To test the interobserver reliabilities for the primary outcome, the intraclass correlation coefficient with two-sided 95% confidence intervals was calculated. Two investigators (TH and ST) separately measured the velocity of blood flow in the profunda femoris for 10 limbs of healthy volunteers.

All statistical analyses were performed with *R* (the *R* Foundation for Statistical Computing).

## 3. Results

**Figure 2:**
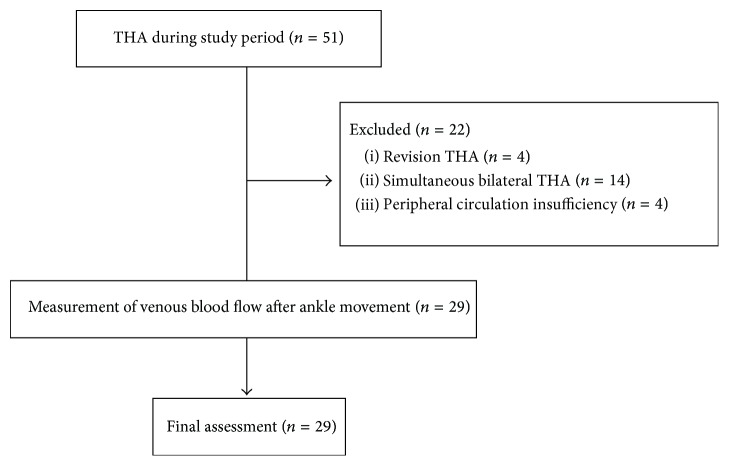
Participant flow chart. THA, total hip arthroplasty.

A total of 51 patients underwent THA during the study period and were eligible for inclusion in the study. The flowchart presented in Figure 2 outlines the study. Four patients that underwent revision THA, seven patients (14 hips) that underwent simultaneous bilateral THA, and four patients that had peripheral circulation insufficiency in the lower limb were excluded. Thus, 29 patients were finally included in the study.  Table 1 summarizes the demographic characteristics of the patients. No deep vein thrombosis was found one day after surgery, and no symptomatic pulmonary embolism occurred.

**Figure 3:**
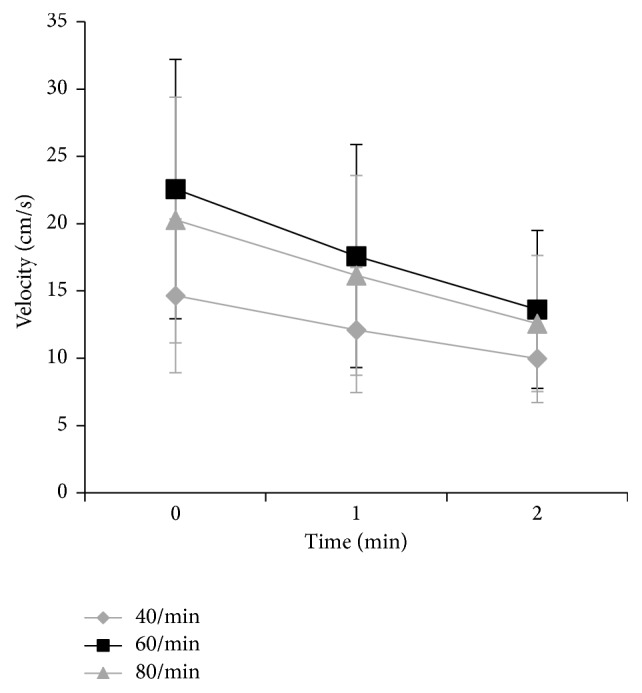
Velocity of blood flow in the profunda femoris after active ankle movement prior to total hip arthroplasty (mean ± standard deviation). The velocity of venous blood flow after active ankle movement at 60 contractions per minute was significantly faster than those after active ankle movement at 40 and 80 contractions per minute (*p* = 0.0001, repeated-measures ANOVA). The horizontal line indicates the time after active ankle movement.

In the baseline velocity of venous flow in the profunda femoris measured one day before surgery, the velocity after active ankle movement at 60 contractions per minute was significantly faster than those after active ankle movement at 40 or 80 contractions per minute (*p* = 0.0001) (Figure 3 and Table 2). In post hoc analysis, all measurements showed significant differences between 60 and 80 contractions per minute (*p* < 0.05) and between 80 and 40 contractions per minute (*p* < 0.05). In all measurements, the velocity was fastest at the end of active ankle movement and thereafter decreased gradually with time.

### 3.1. Primary Outcome

**Figure 4:**
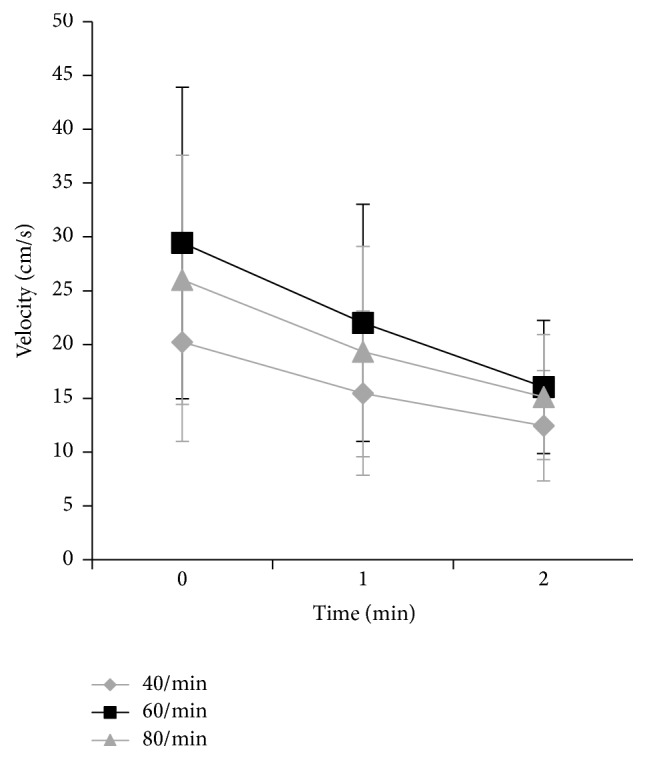
Velocity of blood flow in the profunda femoris after active ankle movement 2 days after total hip arthroplasty (mean ± standard deviation). The velocity of venous blood flow after active ankle movement at 60 contractions per minute was significantly faster than those after active ankle movement at 40 and 80 contractions per minute (*p* = 0.0007, repeated-measures ANOVA). The horizontal line indicates the time after active ankle movement.

At two days after surgery, the mean velocity of venous blood flow in the profunda femoris after active ankle movement at 60 contractions per minute was significantly faster than those after active ankle movement at 40 and 80 contractions per minute (*p* = 0.0007) (Figure 4 and Table 3). In post hoc analysis, all measurements showed significant differences between 60 and 80 contractions per minute (*p* < 0.05) and between 80 and 40 contractions per minute (*p* < 0.05), except for the velocity between 60 and 80 contractions per minute at 2 minutes after finishing active ankle movement (*p* = 0.14).

### 3.2. Interobserver Reliability

As an indicator of interobserver variability, the intraclass correlation coefficient score of the measurement of venous blood flow by two investigators was 0.849 (95% confidence interval, 0.428 to 0.962).

## 4. Discussion

The most important finding of this study was that when active ankle movement was performed at 60 contractions per minute, the mean velocity of venous blood flow in the profunda femoris was faster than those with ankle movement at 40 and 80 contractions per minute in patients that underwent THA.

Mechanical thromboprophylaxis has been considered both safe and effective in patients undergoing THA [4]. Although active ankle movement plays a predominant role in mechanical thromboprophylaxis, the most effective contractions had not been investigated. Although active ankle movement increases muscle blood flow, excessively frequent movement also increases intramuscular pressure [5]. Therefore, the most reasonable frequency of active ankle movement would be difficult to estimate. This was the first study to investigate the impact of active ankle movement frequency on the velocity of lower limb venous flow following THA.

Several limitations should be noted in this study. First, although this study clearly demonstrated that ankle movement at 60 contractions per minute was reasonable to achieve stable venous blood flow, the sample size was underpowered to make definitive conclusions about the prevalence of VTE. Second, as this study was performed in a single hospital, care should be taken when generalizing the findings to the community setting.

With regard to clinical relevance, this study supported the applicability of active ankle movement for preventing VTE following THA. All frequencies of ankle movement investigated in this study increased the velocity of venous blood flow in the profunda femoris and may have ameliorated venous stasis, which plays a central role in the initiation of thrombosis [8]. This study suggested that the active ankle movement at 60 contractions per minute should be recommended after THA.

## 5. Conclusion

Active ankle movement performed at a frequency of 60 contractions per minute provided a faster mean venous blood flow velocity in the profunda femoris compared with that performed at 40 or 80 contractions per minute in patients undergoing THA.

## Figures and Tables

**Table 1:** Patient demographics.

Variable	Value
Gender	
Female	27
Male	2
Age (years)^*∗*^	61.3 ± 11.6 (37–87)
Height (cm)^*∗*^	152.9 ± 7.3 (137–170)
Weight (kg)^*∗*^	55.7 ± 8.5 (45.8–74.4)
Body mass index^*∗*^	23.9 ± 4.2 (17.6–33.7)
Diagnosis	
Osteoarthritis of the hip	28
Osteonecrosis of the femoral head	1
Affected side	
Right	9
Left	20
Preoperative plantar flexion angle (degree)^*∗*^	37.5 ± 4.7 (30–45)
Preoperative dorsiflexion angle (degree)^*∗*^	18.2 ± 2.7 (10–20)
Patient-based evaluation for hip joint disease (point)^*∗*‡^	30.4 ± 16.7 (10–69)
Duration of operation (minute)^*∗*^	64.3 ± 25.9 (37–165)
Intraoperative blood loss (milliliter)^*∗*^	480 ± 275 (200–1700)
Lengthening of lower legs (millimeter)^*∗*^	7.0 ± 6.2 (0.3–22.6)

^*∗*^Values are expressed as means ± standard deviation, with ranges in parentheses.

^‡^The score was evaluated using the Japanese Orthopaedic Association hip disease evaluation questionnaire [9].

**Table 2:** Preoperative mean velocity of blood flow of profunda femoris measured one day before surgery.

	40 contractions/min	60 contractions/min	80 contractions/min	*p*
Just after ankle movement	14.6 ± 5.7	22.5 ± 9.6	20.2 ± 9.1	0.0001^*∗*^
1 minute after ankle movement	12.1 ± 4.6	17.5 ± 8.2	16.1 ± 7.4	
2 minutes after ankle movement	9.9 ± 3.2	13.6 ± 5.8	12.5 ± 5.0	

Values are expressed as means ± standard deviation.

^*∗*^Repeated-measures ANOVA.

**Table 3:** Postoperative mean velocity of blood flow of profunda femoris measured two days after total hip arthroplasty.

	40 contractions/min	60 contractions/min	80 contractions/min	*p*
Just after ankle movement	20.2 ± 9.2	29.4 ± 14.4	26.0 ± 11.5	0.0007^*∗*^
1 minute after ankle movement	15.4 ± 7.6	22.0 ± 11.0	19.3 ± 9.7	
2 minutes after ankle movement	12.4 ± 5.1	16.0 ± 6.1	15.1 ± 5.8	

Values are expressed as means ± standard deviation.

^*∗*^Repeated-measures ANOVA.
